# Development of a Quantitative FMRP Assay for Mouse Tissue Applications

**DOI:** 10.3390/genes12101516

**Published:** 2021-09-26

**Authors:** Tatyana Adayev, Giuseppe LaFauci, Weimin Xu, Carl Dobkin, Richard Kascsak, W. Ted Brown, Jeffrey H. Goodman

**Affiliations:** 1 New York State Institute for Basic Research in Developmental Disabilities, New York, NY 10314, USA; pippo51@aol.com (G.L.); weimin.xu@opwdd.ny.gov (W.X.); dobkincx@gmail.com (C.D.); rrkascsak@msn.com (R.K.); wtbibr@aol.com (W.T.B.); jeffrey.goodman@opwdd.ny.gov (J.H.G.); 2 Perkins Center, University of Sydney Camperdown, Sydney, NSW 2006, Australia

**Keywords:** fragile X syndrome, FXS, FMRP, quantitative assay, mouse tissue, C57BL/6J, FVB, development, seizure threshold, dried blood spots

## Abstract

Fragile X syndrome results from the absence of the FMR1 gene product—Fragile X Mental Retardation Protein (FMRP). Fragile X animal research has lacked a reliable method to quantify FMRP. We report the development of an array of FMRP-specific monoclonal antibodies and their application for quantitative assessment of FMRP (qFMRPm) in mouse tissue. To characterize the assay, we determined the normal variability of FMRP expression in four brain structures of six different mouse strains at seven weeks of age. There was a hierarchy of FMRP expression: neocortex > hippocampus > cerebellum > brainstem. The expression of FMRP was highest and least variable in the neocortex, whereas it was most variable in the hippocampus. Male C57Bl/6J and FVB mice were selected to determine FMRP developmental differences in the brain at 3, 7, 10, and 14 weeks of age. We examined the four structures and found a developmental decline in FMRP expression with age, except for the brainstem where it remained stable. qFMRPm assay of blood had highest values in 3 week old animals and dropped by 2.5-fold with age. Sex differences were not significant. The results establish qFMRPm as a valuable tool due to its ease of methodology, cost effectiveness, and accuracy.

## 1. Introduction

The fragile X syndrome (FXS) (OMIM 309550) is an X-linked disorder characterized by intellectual, learning, and behavioral disabilities and is the most common genetic cause of autism [1,2,3]. The hallmark of the disorder is the absence or drastic reduction of the protein FMRP (fragile X mental retardation protein), which is essential for normal neural development [4]. FMRP functions to inhibit the translation of numerous mRNAs by binding to mRNAs in polysomes. It is encoded by the fragile X mental retardation gene 1 (*FMR1*) which is highly conserved and consists of 17 exons spanning 38 kb of the X chromosome at Xq27.3 [2,5]. Alternative splicing of *FMR1* may produce at least 24 predicted isoforms of FMRP [6,7,8]. However, the physiological roles played by these isoforms are diverse and ever-expanding [9,10,11]. With the exception of rare deletions and intragenic *FMR1* mutations [12,13], in most FXS cases, the loss of FMRP is caused by a CGG triplet repeat expansion in the 5′ untranslated region of exon 1 of *FMR1* to more than 200 repeats, which is considered a full mutation (FM). This expansion causes hypermethylation of the promoter and silences *FMR1* transcription [4,13,14,15]. Normal *FMR1* alleles, which contain 6 to 44 CGG repeats, are stable and only rarely change repeat number on transmission. *FMR1* alleles with an intermediate number of CGG repeats (45 to 54) sporadically show a low level of instability [16,17]. Individuals with alleles containing 55 to 200 CGG repeats are considered to have a premutation, and may have decreased FMRP levels associated with augmented *FMR1* mRNA [18] that is inefficiently translated [19,20]. Premutation alleles are unstable and can expand to the full mutation upon maternal transmission with a rate that is dependent upon the number of CGG repeats and AGG interruptions [17].

Currently, the laboratory diagnosis of fragile X syndrome is based on DNA analysis that determines the number of CGG repeats and/or the degree of methylation of *FMR1*. DNA samples are amplified by PCR and analyzed for CGG triplet repeat number, along with Southern blot analysis for clinical diagnosis. Newer methods have also been developed including mass spectrometry-based methods for analysis of PCR digests or a methylation specific-quantitative melt analysis [21,22,23,24,25,26]. However, not all FM patients exhibit the same level of developmental, intellectual, or functional disability. In FM males the comorbidity of autism is approximately 50% and of epilepsy is 10–20%, and intellectual disability ranges from mild to severe [27]. This variability in deficit can be due to somatic mosaicism in repeat size or the degree of hypermethylation of *FMR1* at the cellular level, or other factors, such as variable modifier genetic effects. Thus, while DNA analysis of the blood is sufficient for a clinical diagnosis, alternative methods are required to determine the role of FMRP in brain development and function. We previously developed quantitative FMRP methods for human studies [28,29,30] that allow for an in-depth look at genotype-phenotype correlates [31]. Due to the limitations of studying the function of FMRP in human tissue, mouse models are widely used, and well suited for a determination of the role of FMRP in brain development and related pathological conditions.

The establishment of immunoassays for the direct quantification of FMRP is one of the indispensable tools for a better understanding of the functional roles of FMRP. In a quest to generate highly specific FMRP assessment tools, we developed an array of anti-FMRP monoclonal antibodies (mAbs) using recombinant mouse and human FMRP as immunogens. Here, we report the development of an FMRP capturing immunoassay using mAb5C2, which binds with high avidity to both human and mouse FMRP. We used this Luminex-based assay (qFMRPm) to quantify and compare the variability of FMRP expression across brain structures of six mouse strains. Additionally, we examined age-dependent changes in CNS FMRP expression in two selected mouse stains, C57BL/6J and FVB, and explored the possibility of FMRP involvement in developmental seizure threshold differences between C57Bl/6J and FVB. Finally, we tested the assay performance on mouse dried blood spots (DBS) as a sample source. The results suggest that this assay can be useful in studying the developmental, physiological, and tissue-specific variation of FMRP expression in mouse models including gene therapy applications.

## 2. Materials and Methods

### 2.1. Animals

All animals used in this study were maintained in an OLAW accredited Animal Facility according to protocols approved by the Institutional Animal Care and Use Committee. Different strains of mice were used in the following procedures or assessments: (1) FMRP mAb production—BALB/c (*n* = 3) and *FMR1* KO FVB *Fmr1*^tm1Cgr^ (*n* = 3). (2) Determination of interstrain variability of FMRP expression in the brain—C57BL/6J, FVB/J, BTRB, SAMR1, SAMP10 and CD-1 adult male mice (*n* = 3 of each). (3) Developmental expression of FMRP at 3, 7, 10, and 14 weeks of age in the brain—male C57BL/6J (*n* = 27) and FVB/J (*n* = 18) mice; and blood—male (*n* = 10) and female (*n* = 10) C57BL/6J. Timed-pregnant dams (four dams for each strain) were purchased from Jackson Laboratory (Bar Harbor, ME, USA). (4) Determination of the threshold in pilocarpine-induced Status Epilepticus (SE)—3 week old non-littermate males C57BL/6J (*n* = 22) and FVB/J (*n* = 25).

### 2.2. Antibodies

The array of anti-FMRP mouse monoclonal antibodies against mouse and human FMRP was generated in-house as described below. Anti-FMRP MAB2160 (clone1C3) and anti-GAPDH monoclonal antibody MAB374 were purchased from Millipore Sigma, (EMD Millipore, Billerica, MA, USA). In-house produced, rabbit anti-FMRP polyclonal antibody R477 was previously described [28,29]. Goat anti-rabbit phycoerythrin conjugated IgG (P2771MP) was purchased from Invitrogen (Carlsbad, CA, USA). The anti-GST sc-138 antibody was purchased from Santa Cruz Biotechnology, Inc. (Dallas, TX, USA).

### 2.3. Expression and Purification of FMRP Antigen

A clone carrying a 1.8 kb mouse cDNA (Mc 2.17; S. Warren) containing the entire *Fmr1* ORF and the 3′ untranslated region (courtesy of Robert Denman) was adapted for directional cloning into the vector pENTR/D-TOPO (Invitrogen, Waltham, MA, USA). The forward primer (5′*CACC*-ATGGAGGAGCTGGTG3′) included a 4 base pair sequence (italic) on the 5′ end which allows directional cloning into the pENTR vector. The reverse primer (5′TACTCCATTCACCAGCGG3′) encompassed the 3′ end of the ORF without the termination codon, thus allowing the in-frame expression of the downstream vector sequences (V5 epitope and 6xHis tag). PCR was performed using Pfu Ultra Hotstart DNA polymerase (Stratagene, La Jolla, CA, USA) and the product was cloned into the pENTR vector using the TOPO cloning protocol. A clone carrying the m*Fmr1* ORF, pENTR-mFX was used to transfer the *Fmr1* insert to a linear recombinant baculovirus DNA (BaculoDirect C-Term) harboring *LacZ* and the Herpes Simplex Virus type 1 thymidine kinase (HSV1 *tk*) genes (BaculoDirect Baculovirus Expression System; Invitrogen). The products of the in vitro recombination were introduced into Sf9 insect cells using a lipid-mediated transfection system (Cellfectin; Invitrogen). Non-recombinant baculoviruses expressing the HSV1 *tk* were selectively eliminated by adding ganciclovir to the Sf9 culture medium. Recombinant, FMRP-expressing baculoviruses were used to prepare high titer stocks. The nickel-nitrilotriacetic acid column chromatography (Ni-NTA columns, Ni-NTA system; Invitrogen) purification of the recombinant FMRP was performed according to the company’s protocol. Ni-NTA column fractions (1 mL each) were analyzed by Coomassie Blue and Western blot (WB) using the anti-FMRP mAb2160.

A baculovirus expressing human FMRP in frame with a 6xHis tag was obtained from M. Toth [32]. This baculovirus was used to infect Sf9 insect cells as described above for mouse FMRP and followed the same protocol for Ni-NTA purification of the recombinant human FMRP as described above.

### 2.4. Immunization of Mice and Generation of Hybridomas

Three 7 week old BALB/c female mice were immunized four times by subcutaneous injection of 100 µg of purified recombinant mouse FMRP in complete Freund’s adjuvant (Sigma Corp., St. Louis, MO, USA) at 2–3 week intervals. The array of mAb against human FMRP was produced as previously described for mAb 6B8 [28]. Briefly, human FMRP in TiterMax adjuvant (CytRx, Norcross, GA, USA) was used to immunize three 7 week old FVB *Fmr1*^tm1Cgr^ male mice by a four 100 μg subcutaneous injection regiment at 3 week intervals. In all instances, retro-orbital bleeds were tested for anti-FMRP antibodies by ELISA using 96 well plates coated with 5 µg/ml of recombinant antigen diluted in carbonate buffer [33]. The highest responding mouse in each group was chosen for hybridoma generation and received additional injections of 50 μg of the immunogen in PBS for three consecutive days prior to sacrifice. The NSO myeloma line (ATCC, Manassas, VA, USA) served as the fusion partner for the splenocytes. Following PEG fusion, cultures were processed as described previously to clone hybridomas producing anti-FMRP mAbs [33,34]. The anti-FMRP mAbs were isolated and purified from ascites fluid by protein-G spin column chromatography (Pierce kit Cat. # 89979) and isotyped according to the manufacturer’s instructions (Pierce Kit Cat. # 26179, Thermo Fisher Scientific, Waltham, MA, USA).

### 2.5. Epitope Mapping

#### Recombinant FMRP Fragment Library

To epitope map the FMRP mAb we designed a recombinant FMRP (recFMRP) fragment library using a set of *FMR1* primers (listed in Table 1) containing restriction sites for unidirectional cloning of PCR products into pGEX-4T-1 plasmid (Invitrogen). Primers for domain 7 (R7) carried XhoI site (forward primer) and EcoRI site (reverse); all the others were engineered with Bam HI (forward) and EcoRI (reverse) sites.

As a template, we used pCR-hFMR1 carrying a *FMR1* ORF without exon 12; for FMRP fragments X12, 4X, and 4, we used pET21-hFMR1 carrying a *FMR1* ORF including exon 12 (Table 1). GST-FMRP-R*X* fusion proteins were expressed in DH5α-competent *E. coli* strain by inducing the bacterial culture (at OD600 ~1) with 0.5 mM isopropyl β-D-1- thiogalactopyranoside (IPTG) at 20 °C overnight for protein production. Fusion proteins were purified by glutathione resin as previously described [35], confirmed for expected mobility shift consistent with the sizes of the inserted FMRP, and used for screening of mAb clones by WB.

### 2.6. Pepscan

The tentative linear epitopes of sixteen hybridomas were determined by a study conducted at Pepscan Presto BV (Zuidersluisweg 2, 8243RC Lelystad, The Netherlands). Residues 51 to 500 of human FMRP (Isoform 6; UniProt # Q06787-1) were used to design a library of overlapping 15-mer peptides with an offset of one residue. The synthesis was performed using Pepscan’s proprietary Chemically Linked Peptides on Scaffolds (CLIPS) technology. The antibody binding to each of the CLIPS-coupled peptides was tested in a PEPSCAN-based ELISA, and the color development was quantified with a camera and an image processing system [36,37].

### 2.7. Preparation of the Mouse Tissue, Cellular, and DBS Extracts

Mice were anesthetized by 1–4% isoflurane inhalation 80% N_2_: 20% O_2_ gas mixture and decapitated with a guillotine. Brains were removed and used as whole or further dissected for the neocortex, hippocampus, brainstem, and cerebellum, weighed, and snap frozen on dry ice. Tissue homogenates were prepared by lysis of roughly minced tissue in 5 volumes of cold homogenizing buffer (10 mM HEPES, pH 7.4, 200 mM NaCl, 0.5% Triton X-100, 30 mM EDTA, 1/10 dilution of Complete Mini Protease Inhibitor Cocktail; Roche) and transferred into an ice-cold Dounce homogenizer. Tissue was homogenized on ice, pulse-sonicated in a Branson digital sonifier for 15 s, and centrifuged for 30 min at 16,000× *g* at 4 °C. Supernatants were stored at −70 °C.

The long-term lymphoblastoid (LTL) cell lysates were prepared from cultures established from samples of fragile X patients or normal *FMR1* genotype individuals as previously described [28]. Cellular extracts were prepared as described for tissue lysates and stored frozen at −70 °C. The protein concentration of the lysates was determined with a Pierce BCA Protein Assay Kit (Thermo Fisher Scientific, Waltham, MA, USA) and used for SDS-PAGE and WB.

Blood was spotted onto ID bloodstain cards (WB100014, GE Healthcare, Life Sciences), dried, and stored as previously described [28]. Three millimeter DBS punches were extracted with 50 μL M-PER buffer supplemented with Complete Mini Protease Inhibitor Cocktail (Roche), antipain and chymostatin in 96 well multiscreen plates. Following a 3 h extraction at room temperature, the eluates were collected by centrifugation at 2000× *g* for 10 min and 10–20 μL of the extract was used per assay well for FMRP quantification.

### 2.8. SDS-PAGE and Western Blot Analysis

Purified recombinant FMRP, mouse brain homogenates, lymphoblastoid cell lysates, and *E. coli* lysates expressing domains of FMRP were prepared as described above and separated on Criterion 4–15% polyacrylamide Tris-HCl gels (Bio-Rad Laboratories, Hercules, CA, USA) at 200 mV for 1 hour according to the manual. The gels were either stained with Coomassie blue or transferred onto 0.22 μm PVDF membranes (Bio-Rad Laboratories) and blotted as previously described [28].

### 2.9. Luminex Assay Procedure for Mouse FMRP Quantification (qFMRPm)

Capturing monoclonal antibody, mAb 5C2, was coupled to 5 × 10^6^ xMAP Microplex microspheres (Luminex Corporation, Austin, TX, USA) according to the manufacturer’s instructions. qFMRPm assays were prepared in a 96 well format analogous to previously described human FMRP quantitative assay qFMRP [28,29]. Each reaction well was set to include a total volume of 100 μL and included either diluted GST-MR7 standard or 3–10 μg of protein lysate/homogenate sample diluted to 50 μL and 3000 mAb 5C2-coupled microspheres resuspended in 50 μL assay buffer (60 beads/μL). During the analyte capture step, reaction plates were incubated in the dark, and shaken for 6 hours at room temperature. A vacuum manifold (Multiscreen™ HTS Vacuum Manifold kit, Millipore Corporation, Billerica, MA, USA) was used to remove the supernatant and wash microspheres. For detection, anti-FMRP R477 rabbit polyclonal antibody was diluted to 1.6 μg/mL (1:625 dilution) in assay buffer and 100 μL per assay well was applied at 4 °C overnight. The microspheres were washed and incubated with goat anti-rabbit IgG conjugated to phycoerythrin (100 μL, 2 μg/mL) for two hours at room temperature with agitation. Finally, the microspheres were resuspended in 100 μL assay buffer, and analyzed (in duplicate or triplicate) using the Luminex-200 system (Luminex Corporation, Austin, TX, USA). The xPONENT software was used for the mean fluorescent intensity (MFI) value analysis. Any sample with a coefficient of variance above 15% was repeated to achieve acceptable values.

### 2.10. Recombinant Fusion Protein for qFMRPm

A glutathione S-transferase (GST) fusion protein carrying the epitopes of both mAb 5C2 and R477 was constructed in two steps similar to previously described qFMRP standard GST-SR7 designed for quantitative assay of human FMRP [28]. In brief, a double-stranded oligomer (5′ to 3′-GATCCAGGGTTGGACCTAATGCCCCAGAAG and CTTCTGGGGCATTAGGTCCAACCCTG) encoding a 10 amino acid sequence of FMRP (residue 344 to 353), including the mAb 5C2 immunoreactive region, was cloned into vector pGEX-4T (GE Healthcare) via BamHI and EcoRI insertion. The resulting plasmid was modified through EcoRI and XhoI cloning to include the portion of *FMR1* coding for amino acids 546 to 605 of the protein recognized by anti-FMRP R477 rabbit polyclonal antibody (R477). The latter sequence was obtained by PCR using a cloned *FMR1* cDNA and forward/reverse primers for region R7 listed in Table 1. A plasmid of pGEX-hFMR1-MR7 was first transformed into *E. coli* DH5α cells, and colonies were IPTG induced and screened for dual recognition by mAb5C2 and R477. For large stock protein production, the construct was expressed in *E. coli* strain BL21. Following the IPTG induction, the fusion protein, GST-MR7, was purified as described for GST-SR7, aliquoted, lyophilized, and stored at −70 °C.

Dilutions of GST-MR7 were used to generate a 7-point standard curve ranging from 1.56 to 100 pmol/L, where MFI was plotted against the concentration. The amount of FMRP in tissue lysates was reported either as pmol/L concentration or recalculated to reflect a mass of FMRP per unit of total protein content (FMRP, 10^−12^ g/10^−6^ g of lysate) using the following formula:

(1) $$\mathrm{Mass}\;\left(g\right)=\frac{\mathrm{Concentration}\;\left(\mathrm{mol}/L\right)\times \;\mathrm{Volume}\;\left(L\right)\times \;\mathrm{Molecular}\;\mathrm{Weight},\;\mathrm{MW}\;\left(g/\mathrm{mol}\right)}{\mathrm{amount}\;\mathrm{of}\;\mathrm{the}\;\mathrm{sample}\;\mathrm{load}\;\left(g\;\mathrm{or}\;L\right)}$$

where Concentration is the value obtained in qFMRPm assay; Volume is the reaction volume per assay well, and MW is taken as 72,000 g/mol, which corresponds to the major FMRP band recognized by mAb 5C2 in WB; amount of the sample load is either the amount of total lysate per reaction well based on the protein estimation or the unit volume of the whole blood per DBS punch. A 3 mm punch was determined to contain 2.1 μL of blood.

### 2.11. Pilocarpine-Induced Status Epilepticus (SE)

Non-littermate male C57BL/6J and FVB/J mice at 3 weeks of age were pretreated with intraperitoneal injection (i.p.) of atropine methyl bromide (1 mg/kg) for 30 minutes followed by pilocarpine hydrochloride (250–365 mg/kg, i.p.). Each animal was observed to determine the onset of behavioral seizure activity. The motor seizure scoring system of Racine scale was used to determine the onset of seizure activity. Animals that exhibited continuous behavioral seizure activity for more than 5 min were considered to be in SE. One hour after the onset of SE, each animal received an injection of diazepam (5 mg/kg, i.p.). The threshold for the induction of SE was determined as an effective dose (ED_50_) for the minimum dose of pilocarpine required to induce SE in at least 50% of the animals tested, with at least an equivalent percentage of animals surviving the treatment for each strain.

## 3. Results

### 3.1. Generation of an Array of Anti-FMRP Antibodies 

**Figure 1:**
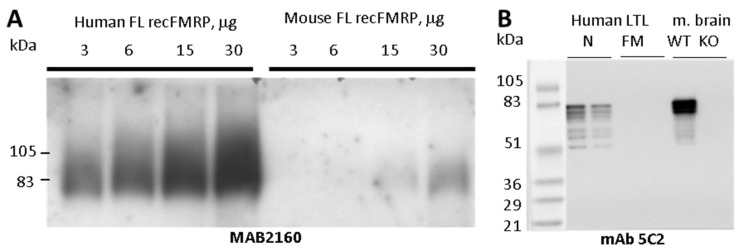
FMRP immunogen characterization and anti-FMRP mAb5C2 immuno-reactivity. (**A**) Purified recombinant full length (FL) human and mouse FMRP loaded at 3, 6, 15, or 30 μg per lane and resolved on 4–15% Criterion Tris-HCl gel and blotted with MAB2160 (clone 1C3). (**B**) mAb5C2 WB analysis of human LTL lysates from normal (N) and full mutation (FM) *FMR1* genotype males (30 and 15 μg per lane per genotype) and mouse brain lysates from a wild type (WT) and Fmr1 KO mouse (KO) 15 μg each.

To develop a high affinity FMRP specific monoclonal antibody, we purified full length recombinant FMRP from baculovirus-infected Sf9 insect cells. Recombinant mouse and human FMRP were purified under denaturing conditions on Ni-NTA columns as described in the Methods section. Both recombinant proteins were recognized in Western blots (WB) by well-characterized commercial anti-FMRP antibody MAB2160. When the two antigens were examined side-by-side, MAB2160 reactivity to the human antigen was at least twenty times higher than that of the mouse antigen (Figure 1A). Because MAB2160 was raised against a full-length human FMRP, this difference in species-specificity was not expected.

Mice were immunized with the recombinant human or mouse FMRP. Hybridoma were produced and screened for reactivity against recombinant immunogen, fragile x related protein 1 (FXR1P) and fragile x related protein 2 (FXR2P) by ELISA. Only FMRP-positive clones that lacked immunoreactivity to FXR1P and FXR2P were isolated, purified, and used for ascites production. The antibodies (all isotyped as IgG1K) were analyzed by WB for reactivity to the recombinant FMRP fragment library, human lymphoblastoid cells, and mouse brain homogenates. The 19 clones with positive FMRP recognition are listed in Table 2.

### 3.2. Antibody Reactivity with Human and Mouse Tissues and Epitope Mapping

All the antibodies obtained using mouse FMRP as an immunogen reacted with both human and mouse FMRP. Some antibodies against human FMRP immunogen were specific for human FMRP (clone 6B8 was previously described, specificity is shown in Supplementary Figure S1D) [28]. The antibodies mAb5C2 (Figure 1B) and mAb1B12 (Supplementary Figure S1B,E), raised against mouse FMRP, had the highest affinity to endogenous mouse FMRP. In most cases, the antibodies detected three broad bands (68 to 80 kDa) in both the human and mouse samples. However, a stronger signal was usually obtained with the mouse antigen. No signal was detected with lymphoblastoid cells derived from male FM FXS patients or from Fmr1 KO mouse brain (Figure 1B). In normal human lymphoblastoid cells, most of the antibodies (11/14), including mAb5C2 and mAb1B12, stained an additional three bands migrating with apparent molecular weight ranging from 51 to 61 kDa, which were not evident in mouse brain.

To locate the antibodies’ binding sites, we constructed a panel of overlapping GST-fusion recFMRP proteins each carrying a fragment of FMRP (Methods, Table 1). These fusion proteins were used to screen the antibodies by WB. As shown in Table 2 (last column), this approach allowed us to determine, for each antibody, the region of FMRP that contained the antibody recognition site. For example, mAb2G5 reacted with both clones R1A and R2, indicating that its binding site must be the sequences shared by the two clones (aa76–132 coded by exons 4 and 5). A large group of antibodies comprising mAb5C2 and mAb1B12 reacted to sequences of exon 11 (clones R4, R4M and R4). The reactivity of these antibodies to fragments A2, and Linker, each carrying short overlapping domains of exon 11, allowed us to establish that the sequence _348_NAPEE_352_ is essential for binding (Table 2).

**Figure 2:**
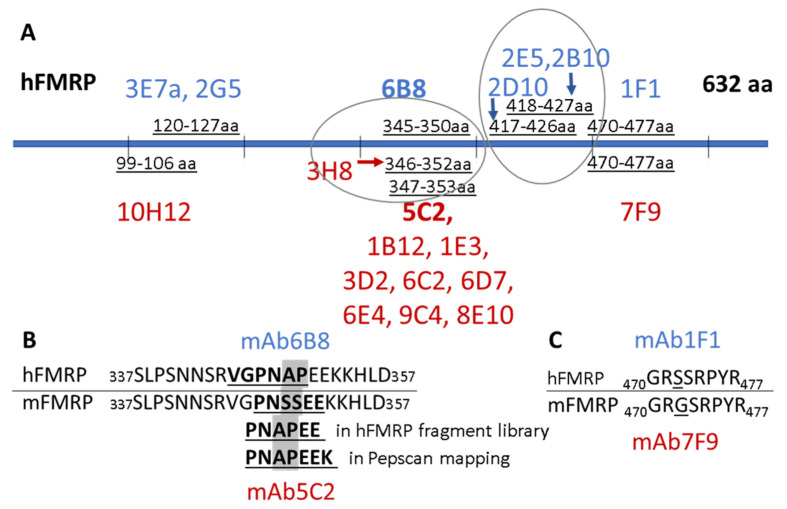
Schematic map of the anti-FMRP mAb epitopes. The epitopes for mAb clones are shown in (**A**) with anti- hFMRP mAbs shown above the schematic view of the protein in blue and anti-mFMRP mAb clones shown below in red. Magnified sequence of super immunogenic region and epitopes of mAb5C2 and mAb6B8 (**B**) and mAb1F1 and mAb7F9 (**C**).

An adjacent and overlapping sequence _345_VGPN_348_ was found to be the core sequence for binding of mAb6B8. Two adjacent amino acids _349_AP_350_ influence the binding of mAb6B8 because the antibody does not react to the corresponding mouse FMRP sequence _345_VGPNSS_350_. We also determined the regions of FMRP (Table 2 and Figure 2A) that contain the binding sites of mAb 10H12 (exon 5), mAb 1F1 and mAb 7F9 (exon 14 to 15), and mAb 2D10 (exons 13 to 14). The linear epitopes for 16 of the antibodies described in this study were also determined by Pepscan analysis (Table 2, third column, and Supplementary Materials Report S1), confirming and detailing the data from recFMRP fragment analyses.

The collective immunoreactivity map for all mAb clones is shown in Figure 2A. Nine antibodies (clones 1E3, 1B12, 5C2, 3D2, 6C2, 6D7, 6E4, 9C4, and 8E10) were Pepscan-tested under stringent conditions and all avidly bound peptides with core sequence _347_PNAPEEK_353_ located in exon 11 (Table 2). This core embraces the sequence _348_NAPEE_352_ found to be essential for binding in WB of mAb1E3, mAb3D2, mAb1B12, and mAb5C2 (Table 2). This region was dominant for immunogenicity toward mouse FMRP. This group of antibodies reacted in WB with both mouse and human FMRP, but had higher avidity to the mouse sequence _347_PNASSEEK_353_ where two amino acids _349_AP_350_ are replaced with _349_SS_350_ (Figure 2B).

The linear epitopes from Pepscan analysis for human FMRP-derived antibodies mAbs 2D10, 2E5, and 2B10 shared the same epitope on exon 13 (with the exception of one residue shift in 2D10 epitope) (Figure 2A). The epitope _417_LDYHLNYLKEV_427_ coded by exon 13 is conserved between human and mouse but was clearly favored in antibody production to human FMRP immunogen. We did not identify an equivalent clone that resulted from the mouse immunized with mouse FMRP. The epitope was shared by mAb2G5 and mAb3E7a and the epitope of mAb10H12 coded by exon 5 and conserved between the sequences of mouse and human FMRP. The immunoreactivity of mAB10H12 is shown in Figure S1.C By contrast, the epitope shared by both mAb1F1 and mAb7F9 (exon 14) has one amino acid change between the two species, _470_GR**S**SRPYR_477_ in human, and _470_GR**G**SRPYR_477_ in mouse (Figure 2C).

### 3.3. Immunoassay for Quantification of Mouse FMRP

**Figure 3:**
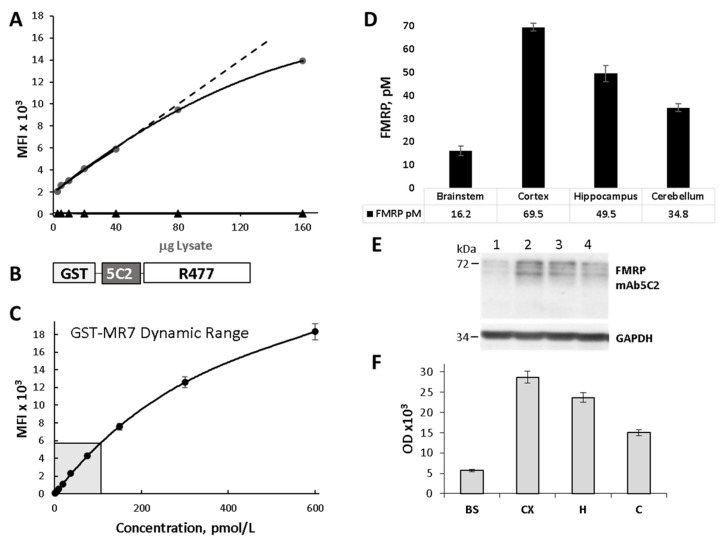
Quantitative FMRP assay for mouse tissue. (**A**) Luminex assay detection of FMRP in wild type (WT) and Fmr1 KO (KO) whole brain lysates. Linear response of the mAb5C2-R477 assay to increasing amounts of WT brain lysate (**circles**) up to 40 μg; Fmr1 KO lysates (triangles) showed only background fluorescence up to 160 μg. (**B**) Schematic view of the abbreviated FMRP standard, GST-MR7, engineered to include the mAb5C2 and R477 epitopes. (**C**) Dynamic range of GST-MR7 tested up to 600 pmol/L showed linearity from 0 to 150 pmol/L. Mean fluorescence intensity MFI = 51.104 pmol/L + 120.98. *R*^2^ = 0.9951. GST-MR7 working range was selected from 0 to 100 pmol/L (shaded box). (**D**) Quantification of FMRP by Luminex-based capture immunoassay, qFMRPm, in lysates from four brain regions of C57BL/6J mice. (**E**) Western blot analysis of samples (10 ug per lane) shown in (**D**) 1. brainstem (BS), 2. cortex (CX), 3. hippocampus (H), 4. cerebellum (C) blotted with mAb5C2 at 1:2000 (upper panel) and anti-GAPDH MAB374 1:30,000 (lower panel). (**F**) Denstometric scan of the WB shown in (**E**).

Our laboratory previously developed a Luminex-based capture immunoassay, qFMRP, that accurately quantifies FMRP using the human-specific mAb6B8 (available through Biolegend, San Diego, CA, USA) capturing antibody [28]. The mAb5C2 (available through Biolegend, San Diego, CA, USA) has high avidity for mouse FMRP and was successfully used for immunohistochemical characterization of FMRP expression in neurons and glia in mice [38,39]. We selected this antibody to establish a parallel Luminex-based immunoassay (qFMRPm) for quantification of FMRP in mouse-derived tissue samples. In the assay, we used mAb5C2-coupled microspheres to capture FMRP, which was followed by detection with the anti-FMRP rabbit antibody R477. First, we evaluated the capacity and specificity of the 5C2-R477 antibody pair to capture and detect endogenous FMRP in the immunoassay of whole brain homogenates from wild type (WT) and *Fmr1* knockout (KO) mice. We used 2.5 to 160 μg of the total protein. The amount of FMRP detected in WT lysates was proportional to the amount of sample load (Figure 3A) and showed linearity up to 40 μg of lysate (*R*^2^ = 0.9945). The KO lysates had a background level of fluorescence in all wells irrespective of load up to 160 μg.

The fusion protein GST-MR7 carrying the epitopes of both mAb5C2 and R477 was used as the protein standard (Figure 3B). We quantified mouse FMRP in 10 μg homogenates of the brainstem, cortex, hippocampus, and cerebellum prepared from tissue of 7 week old C57BL/6J male mice (Figure 3D). FMRP expression was higher in the cortex (69.5 pM) and hippocampus (49.5 pM), and lower in the cerebellum (34.8 pM) and brainstem (16.2 pM). In parallel, we assessed the FMRP content in the same samples by traditional WB with mAb5C2 (Figure 3E,F). The WB was scanned, and the OD of the four brain structures was normalized using GAPDH values (Figure 3E). The FMRP-specific OD was plotted (Figure 3F) and showed FMRP expression similar to that obtained with capture immunoassay. qFMRPm assay was performed in 96 well format in triplicate, and highly correlated with WB-derived OD values for FMRP (Pearson correlation coefficient, *R* = 0.9854).

### 3.4. Interstrain Variation in FMRP Expression in Mice (Six Strains at 7 Weeks of Age)

To study the variation in FMRP expression in mice, we used qFMRPm to quantify the protein in CNS structures of five inbred mouse strains: C57BL/6J, FVB/NJ, BTBR, SAMR1, and SAMP10, and one outbred CD-1 mouse strain. The choice of mouse strains was based on a number of parameters: (1) representing various groups of the inbred mouse family tree [40]; (2) relevance to FX and autism field; (3) distinct synaptic or brain structure-specific phenotype related to deficits in learning and memory. Thus, C57BL/6J (group 4) and FVB/NJ (group 2) were selected for their popularity as background strains for mouse models of FXS and Fmr1 transgenics. BTBR (group 5) is commonly used as a behavioral model for autism. SAMR1 and SAMP10 mouse strains were developed on AKR/J background (group 1), as implied by the names: Senescence-Accelerated Mouse Resistant (SAMR1) or Prone (SAMP10). The latter is a model of brain aging where animals developed diffuse atrophy in the cerebral neocortex with advancing age, and the frontal cortex was most severely affected [41]. For this analysis, we used lysates from four brain structures: the brainstem, cortex, hippocampus, and cerebellum of seven week old male mice of each strain (Table 3). We found a variable expression of FMRP in all four brain structures. In the analyzed mouse strains, the highest and the least variable mean level of the protein was detected in the cortex (mean 65.7 pM, SD = 4.9), the hippocampus was the most variable among the strains (mean 58.7 pM, SD = 14.3), lower levels were found in the cerebellum (mean 39.4 pM, SD = 5.3), and the lowest levels were detected in the brainstem (mean 24.8 pM, SD = 6.6).

### 3.5. Assessment of the Threshold for Pilocarpine-Induced Status Epilepticus (SE)

It is well established that FVB mice are more susceptible to audiogenic seizures (AGS) than C57BL/6J mice at three weeks of age [42]. The *Fmr1* transgenic mice are more susceptible to AGS, which are related to FMRP dosage [43,44]. The two strains, C57BL/6J and FVB, in our FMRP strain variability study assessed at seven weeks of age showed some difference in FMRP levels in the hippocampus. To determine whether the difference in seizure threshold between the two strains at three weeks of age was unique to audiogenic seizures, we determined the threshold for pilocarpine-induced SE in 3 week old male FVB and C57BL/6J mice. At this age, the minimum dose of pilocarpine that induced SE in FVB mice was 280 mg/kg, i.p., whereas the threshold for pilocarpine-induced SE in C57BL/6J mice was 310 mg/kg, i.p., demonstrating that the strain difference in seizure threshold reported for an audiogenic stimulus was also present for pilocarpine induction of SE (Table 4).

### 3.6. Developmental Expression of FMRP in the Central Nervous System and Blood

#### 3.6.1. CNS

**Figure 4:**
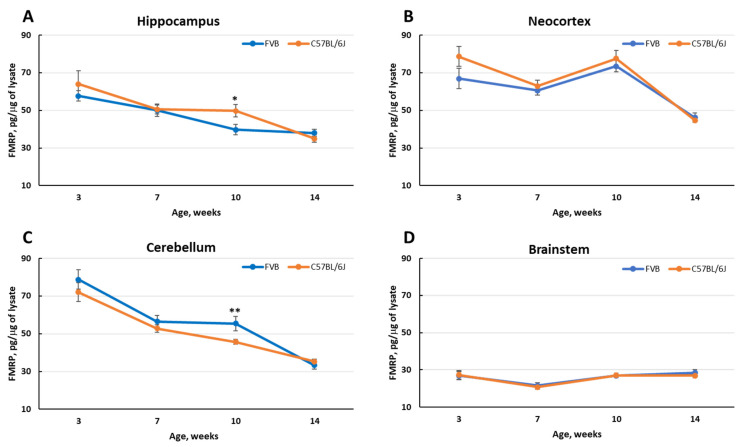
The developmental expression of FMRP in the CNS from 3 to 14 weeks of age in FVB and C57BL/6J mice. The expression of FMRP is illustrated in (**A**). hippocampus; (**B**). neocortex; (**C**). cerebellum, and (**D**). brainstem. Mean presented at 3 weeks of age differential expression of FMRP among the structures: cerebellum > neocortex > hippocampus > brainstem was present in both mouse strains examined. In each strain there was a significant decrease in FMRP expression in both the hippocampus and cerebellum from age 3–14 weeks (*p* < 0.001). A significant decrease in FMRP expression in the neocortex did not appear until week 14 (*p* < 0.001) compared to 3 weeks. At all ages examined the expression of FMRP in the brainstem remained stable. At 10 weeks of age there was a significant difference in the expression of FMRP in the hippocampus (*—*p* < 0.03) and the cerebellum (**—*p* < 0.002) between the strains.

The absence of FMRP can lead to increased neuronal excitability and seizures, as some FXS patients exhibit seizures. FVB mice compared to C57BL/6J mice not only have a lower threshold for audiogenic seizures, but also have a lower threshold for experimentally induced SE. To further explore potential strain differences in the developmental expression of FMRP in the CNS, we used qFMRPm to determine the normal developmental expression of FMRP in the two strains. In an earlier study, mAb 5C2 was successfully used to immunohistochemically profile FMRP expression in the developing brain of C57BL mice [38] at postnatal days 0, 10, and 20, and adults. In our assessment we analyzed four brain regions: the hippocampus, neocortex, cerebellum, and brainstem of C57BL/6J and FVB mice at 3, 7, 10, and 14 weeks of age. To eliminate a possible litter effect, we collected brain tissue from no more than two males per litter per time point. Surprisingly, the post hoc analysis of the data returned a non-significant difference in the CNS expression of FMRP between the two strains at 3 weeks of age across all examined structures (Figure 4), indicating that any difference in audiogenic or pilocarpine-induced seizure threshold reported between the two strains cannot be attributed to a difference in basal FMRP expression. However, we did observe a developmental decrease in FMRP expression in each of the structures examined, except for the brainstem (Figure 4).

#### 3.6.2. Blood

**Figure 5:**
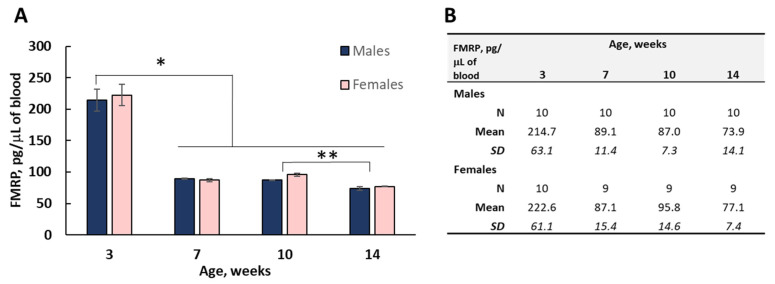
Quantitative assessment of FMRP in DBS extracts derived from C57BL/6J mice. A bar graph representation of the levels of FMRP detected in DBS extracts from male and female mice collected at 3, 7, 10, and 14 weeks of age is shown in (**A**). FMRP presented as gram per unit volume (10^−12^ g/mL) of whole blood as listed in part (**B**). FMRP levels detected in DBS of the 3 week old animals were significantly higher (* *p* < 0.001) compared to the data at 7, 10, and 14 weeks of age. FMRP expression in 14 week old mice vs. 10 week old mice was significantly lower in both sexes (** *p* < 0.05 for males and *p* < 0.01 for females). Error bars represent the SEM of the group.

Because there was no drastic difference in FMRP between C57BL/6J and FVB, for the next set of experiments we used C57BL/6J mice of both sexes. We initiated a blood collection in three week old animals with recurrent blood draws separated by a 3–4 week recovery period at 7, 10, and 14 weeks. Blood was directly spotted onto DBS cards after a tail vein nick, collecting 2–3 free-fallen drops. To eliminate a potential litter effect, we used no more than two animals of both sexes per litter. Known dilutions of a GST-MR7 were used to calculate a standard curve and to determine the amounts of FMRP present in DBS samples (Figure 5). The total volume of blood in younger mice (3 week old group) is physiologically lower and limited the collection volume. FMRP evaluations for all groups were performed with dilutions of the extract from a 3 mm punch to allow multiple readouts. Assays of duplicate extracts or diluted DBS extracts were highly correlated (*r* = 0.96). The FMRP levels reported in Figure 5 are in 10^−12^ gram (pg) of FMRP per unit volume of the blood (μL). No significant difference was found between males and females at any age group (*p* > 0.05, F-Test 0.929). The FMRP expression was highest among 3 week old animals by 2.5-fold. The level of FMRP declined with age and 14 week old animals showed significantly lower levels compared to 10 week old animals (*p* < 0.05 in males and *p* < 0.01 in females).

## 4. Discussion

To successfully use animal models to address questions related to FXS, it is necessary to have the ability to accurately measure FMRP expression in experimental tissue. In this study, we developed an array of mouse monoclonal antibodies against mouse or human FMRP immunogens. Due to the FMRP sequence similarities between species (93%) and the method of the immunogen purification under denaturing conditions, the immune response in mice resulted in the generation of antibodies clustering to four distinct immunogenic regions. These regions are consistent with the FMRP sequence-predicted hydrophobicity plot. For all antibody clones examined, we identified only linear epitopes. We did not identify any conformational antibodies. Production of antibodies to only linear epitopes is likely a consequence of antigen preparation. The denatured form of the immunogen likely prevented the generation of a response to conformational sites on FMRP.

The epitopes of the reported antibodies mapped to the regions of FMRP coded by *FMR1* exons 5, 11, 13, and 14. All FMRP isoforms include exons 5, 11, and 13, and only half of the theoretical FMRP isoforms include exon 14. There is a potential application of our antibodies specific for isoforms with exon 14 (mAb 7F9 and mAb 1F1) for isoform-specific studies.

A super immunogenic region of FMRP coded by exon 11 (Figure 2B), identified by Pepscan and recFMRP fragment library analyses, was recognized by 57% of all antibody clones (or 11 of 19) that resulted from the immunization of two different strains of mice with two different immunogens. For our quantitative assay, we selected the mAb 5C2 clone from this group because of high immunoreactivity to endogenous FMRP, and the location of its epitope within exon 11, a region present in all FMRP splice variants, enabling the capture of all expressed isoforms irrespective of developmental stage. Therefore, the qFMRPm assay is highly sensitive and specific.

The assay permitted an accurate assessment of the variation in the basal levels of FMRP in brain structures of different mouse strains, and the region-specific characteristics. We identified a hierarchy of FMRP expression in different brain regions with the highest expression in the neocortex, followed by the hippocampus, cerebellum, and brainstem. The differences in FMRP expression may reflect differences in glia to neuron ratios in respective regions and potential differences in activity. The overall profile of FMRP expression in different brain structures in mice was consistent with previous semi-quantitative Western blot data reported by Bonaccorso et al. [45].

In 7 week old mice, the hippocampus was the most variable across various mouse strains. This variability in hippocampal expression may be due to differences in litter size and maternal care of the animals used in this assessment. We factored in this possibility in the developmental FMRP expression study, which negated the initial observation of a rather large difference between C57Bl/6J and FVB mice in the hippocampus seen in the intrastrain variability study, but became non-significant in non-littermates of the same age assessed during the developmental expression study.

Because no significant difference in basal FMRP levels was detected in 3 week old mice between the two strains (C57BL/6J and FVB) in the hippocampus and neocortex, the two structures involved in the generation of seizure activity, the lower threshold for seizure susceptibility (in both AGS and pilocarpine-induced models) could not be attributed to strain differences in FMRP expression. Interestingly, all FXS patients have reduced FMRP but not all experience seizures.

We extended the applications of the method to mouse dried blood spots as a tissue source. The FMRP levels in blood exhibit a 2.4- to 2.6-fold drop from values of 214.7 pg/uL in males and 222.6 pg/uL in females at 3 weeks of age, to 89.1 and 87.1 pg/uL (respectively) at 7 weeks of age. The number of white blood cells (WBC) per unit of blood can influence the total amount of FMRP detected. Considering a physiological reduction in WBC number occurring between 3 weeks and 7–10 weeks of life, it is possible that some of the FMRP reduction may be accounted for by the WBC number difference. However, the hematological reference values for C57BL/6 mice at various ages are not uniform [46,47,48] and require a more detailed assessment for the normalization of FMRP on an individual basis. The study was limited by the volume of the collected blood in the youngest group.

This report provides a conceptual framework for the qFMRPm method. It is economical and readily scalable for high throughput applications. The qFMRPm assay is not limited to a tissue lysate that requires animal sacrifice, and is easily performed on dried blood spot extracts. It assays the levels of FMRP directly, which makes the qFMRPm suitable for monitoring the protein expression. Thus, this assay has the potential to be effective in gene therapy applications or treatment surveillance.

## 5. Patents

United States Patent # 8628934 “System and Method for Quantifying Fragile X Mental 1 Protein in tissue and blood samples” was issued on January 14, 2014. The owner of the patent is the Research Foundation for Mental Hygiene, Inc., and G.L., R.K., and W.T.B. are the inventors and declare no conflict of interest.

## Figures and Tables

**Table 1:** Recombinant FMRP fragment library.

Fragment Name	PRIMERS		PCR Product, bp	FMRP Fragment Library, Schematic View
	Forward	Reverse		
R1	GTGGATCCGAGCTGGTGGTGGAAGT	CGGAATTCATCAGGCTGCCAGTTG	127	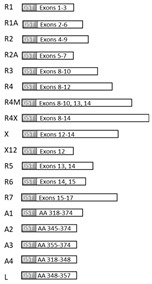
R1A	GTGGATCCAACAACTGGCAGCCTGATAG	CGGAATTCATCCTTATGTGCCGCCTCTT	364	
R2	GTGGATCCCCTTGCTGTTGGTGGTTAG	CGGAATTCCACTGCATCCTGATCCTCTC	607	
R2A	GTGGATCCGATGTGCCAGAAGACTTACG	CGGAATTCAGACAACTTAGTGCGCAGAC	214	
R3	GTGGATCCAAGCAGCTGGAGAGTTCA	CGGAATTCTTCAGCCTCAATCCTCAC	361	
R4	GTGGATCCAGGCAGCTTGCCTCGAGATT	CGGAATTCCTGCCAAGCCTTGAGTTCAG	568	
R4M	GTGGATCCAGGCAGCTTGCCTCGAGATT	CGGAATTCACGCAACTGGTCTACTTC	594	
R4X	GTGGATCCAGGCAGCTTGCCTCGAGATT	CGGAATTCACGCAACTGGTCTACTTC	657	
X	GTGGATCCAGGGTGTTAGTGGCTTCATC	CGGAATTCACGCAACTGGTCTACTTC	187	
X12	GTGGATCCAGGGTGTTAGTGGCTTCATC	CGGAATTCCTGCCAAGCCTTGAGTTCAG	63	
R5	GTGGATCCAGCATCGCTAATGCCACTGT	CGGAATTCCTGTCGCAACTGCTCATCAA	121	
R6	GTGGATCCGAGCAGTTGCGACAGATTG	CGGAATTCTCCACGTCCTCTTCCTCCT	343	
R7	CGGAATTCCGTGGAGGAGGCTTCAA	CCCTCGAGCAGCCGACTACCTTCCACTG	180	
A1	GTGGATCCATTGAGGCTGAAAATGAG	CGGAATTCCTGGACTTTTGTACTGTT	171	
A2	GTGGATCCGTTGGACCTAATGCCCCA	CGGAATTCCTGGACTTTTGTACTGTT	90	
A3	GTGGATCCCATTTAGATATAAAGGAA	CGGAATTCCTGGACTTTTGTACTGTT	60	
A4	GTGGATCCATTGAGGCTGAAAATGAG	CGGAATTCATTAGGTCCAACCCTTGA	93	
Linker (L) oligomer sequence 5′ to 3′ GATCCAATGCCCCAGAAGAAAAAAAACATTTAGATG and 5′ to 3′AATTCATCTAAATGTTTTTTTTCTTCTGGGGCATTG				

The FMRP fragments listed with their designated names, 5′ to 3′ forward and reverse primer sequences, *FMR1* fragment size, and schematic map of the fragment with regards to *FMR1* exon coverage.

**Table 2:** Anti- FMRP mAb clones and corresponding epitopes.

mAb Clone	FMRP Antigen *	hFMRP Epitope (Pepscan) aa	Region of Immunoreactivity (R), aa
10H12†	m	99–106, _99_CDATYNEI_106_	(R6)† 442–540
3H8	m	346–352, _346_GPNAPEEKK_354_	(R4, R4M, R4X), 320–375
1E3	m	347–353 and multiple	(R4, R4M, R4X + multiple), ND
**1B12**, **5C2**	m	347–353, _347_PNAPEEK_353_	(R4, R4M, 4X, A1, A2, L), 347-352, _347_**PNAPEE**_352_
3D2, 6C2, 6D7, 6E4	m	347–353, _347_PNAPEEK_353_	Not tested
9C4	m	Not tested	(R4, R4M, R4X, R6), 320–375, 442–540
7F9	m	470–477, _470_GRGSRPYR_477_	(R6), 442–540
3E7a, 2G5	h	120–127, _120_PATKDTFH_127_	(R1A, R2), 76–132
**6B8**	h	345–350, _345_VGPNAP_350_	(R4, R4M, R4X, A1, A2, A4), 345–350 _345_**VGPN**AP_350_
8E10	h	Not tested	(R4, R4M, R4X), 320–375
2D10	h	417–426, _417_LDYHLNYLKE_426_	(R5, X, R4M, R4X), 409–431
2E5, 2B10	h	418–427 _418_DYHLNYLKEV_427_	Not tested
1F1	h	470–477, _470_GRSSRPYR_477_	(R6), 442–540

* m = mouse; h = human; (R) is region of the hFMRP fragment library as listed in Table 1. Bolded mAb clones had higher affinity toward native FMRP. Bolded portion of an mAb epitopes indicates minimal essential residues in hFMRP sequence. Underlined amino acid indicates a residue difference between human and mouse FMRP. †—mixed clone with initial strong immunoreactivity to R6, was later subcloned and retained only N-terminus immunoreactivity with epitope at aa99–106.

**Table 3:** FMRP detected by qFMRPm in four brain structures of six mouse strains.

Strain	FMRP, (SEM)			
	Brainstem	Cortex	Hippocampus	Cerebellum
C57BL/6J	16.2 (2.1)	69.5 (1.7)	49.5 (3.5)	34.8 (1.8)
FVB/NJ	17.3 (0.9)	69.4 (4.6)	39.8 (2.1)	34.0 (2.5)
CD1	28.6 (0.9)	60.7 (2.8)	58.1 (1.9)	47.3 (1.5)
BTBR	25.3 (1.0)	59.3 (1.6)	55.6 (2.8)	43.3 (1.3)
SAMR1	31.8 (2.0)	70.8 (1.8)	80.2 (2.9)	41.1 (1.0)
SAMP10	29.9 (0.7)	64.6 (2.4)	68.8 (0.7)	35.9 (1.5)
Average	24.8	65.7	58.7	39.4
SD	6.6	4.9	14.3	5.3

The FMRP is reported as pM concentration (10^−12^ mol/L) detected per reaction well.

**Table 4:** Differences in sensitivity to pilocarpine in 3 weeks old C57BL/6J and FVB mice.

Mouse Strain	Dose, mg/kg	% SE	Number of mice Tested	% of Mice Surviving SE
C57BL/6J	310	56	9	100
*n* = 22	320	100	7	29
	365	100	6	0
Threshold value	310	56		100
FVB	250	50	6	67
*n* = 25	260–275	0	9	N/A
	280	100	10	90
Threshold value *	280	100		90

One hour after onset of SE, each animal received an injection of diazepam (5 mg/kg, i.p.), with the exception of mice that died before 60 min of SE. * Although the 250 mg/kg dose met the ED_50_ criteria set forth in the Methods, it was rejected due to inability of higher doses of 260–275 mg/kg to induce SE. Thus, the 280 mg/kg dose was accepted as the SE threshold. Differences in SE induction between C57Bl/6J and FVB mice (*p* = 0.001) were highly significant.

## Data Availability

The data presented in this study are available on request from the corresponding author.

## Supplementary Materials

The following are available online at https://www.mdpi.com/article/10.3390/genes12101516/s1, Figure S1: Western blot analyses and immunoprecipitation studies for select mAb clones; Report S1: Summary of the precision epitope mapping data for anti-mouse and anti-human FMRP antibody clones.

## References

[1] Crawford D.C., Acuña J.M., Sherman S.L. (2001). FMR1 and the Fragile X Syndrome: Human Genome Epidemiology Review. Genet. Med. Off. J. Am. Coll. Med. Genet..

[2] Verkerk A.J.M.H., Pieretti M., Sutcliffe J.S., Fu Y.-H., Kuhl D.P.A., Pizzuti A., Reiner O., Richards S., Victoria M.F., Zhang F. (1991). Identification of a Gene (FMR-1) Containing a CGG Repeat Coincident with a Breakpoint Cluster Region Exhibiting Length Variation in Fragile X Syndrome. Cell.

[3] Richter J.D., Zhao X. (2021). The Molecular Biology of FMRP: New Insights into Fragile X Syndrome. Nat. Rev. Neurosci..

[4] Pieretti M., Zhang F., Fu Y.-H., Warren S.T., Oostra B.A., Caskey C.T., Nelson D.L. (1991). Absence of Expression of the FMR-1 Gene in Fragile X Syndrome. Cell.

[5] Eichler E.E., Richards S., Gibbs R.A., Nelson D.L. (1993). Fine Structure of the Human FMR1 Gene. Hum. Mol. Genet..

[6] Ashley C.T., Sutcliffe J.S., Kunst C.B., Leiner H.A., Eichler E.E., Nelson D.L., Warren S.T. (1993). Human and Murine FMR-1: Alternative Splicing and Translational Initiation Downstream of the CGG–Repeat. Nat. Genet..

[7] Verkerk A.J.M.H., de Graaff E., De Boulle K., Eichler E.E., Konecki D.S., Reyniers E., Manca A., Poustka A., Willems P.J., Nelson D.L. (1993). Alternative Splicing in the Fragile X Gene FMR1. Hum. Mol. Genet..

[8] Sittler A., Devys D., Weber C., Mandel J.-L. (1996). Alternative Splicing of Exon 14 Determines Nuclear or Cytoplasmic Localisation of FMR1 Protein Isoforms. Hum. Mol. Genet..

[9] Bardoni B., Mandel J.-L. (2002). Advances in Understanding of Fragile X Pathogenesis and FMRP Function, and in Identification of X Linked Mental Retardation Genes. Curr. Opin. Genet. Dev..

[10] Davis J.K., Broadie K. (2017). Multifarious Functions of the Fragile X Mental Retardation Protein. Trends Genet. TIG.

[11] Maurin T., Bardoni B. (2018). Fragile X Mental Retardation Protein: To Be or Not to Be a Translational Enhancer. Front. Mol. Biosci..

[12] Quartier A., Poquet H., Gilbert-Dussardier B., Rossi M., Casteleyn A.-S., des Portes V., Feger C., Nourisson E., Kuentz P., Redin C. (2017). Intragenic FMR1 Disease-Causing Variants: A Significant Mutational Mechanism Leading to Fragile-X Syndrome. Eur. J. Hum. Genet..

[13] De Boulle K., Verkerk A.J.M.H., Reyniers E., Vits L., Hendrickx J., Van Roy B., Van Den Bos F., de Graaff E., Oostra B.A., Willems P.J. (1993). A Point Mutation in the FMR-1 Gene Associated with Fragile X Mental Retardation. Nat. Genet..

[14] Coffee B., Ikeda M., Budimirovic D.B., Hjelm L.N., Kaufmann W.E., Warren S.T. (2008). Mosaic FMR1 Deletion Causes Fragile X Syndrome and Can Lead to Molecular Misdiagnosis. Am. J. Med. Genet. A.

[15] Tassone F., Hagerman R.J., Chamberlain W.D., Hagerman P.J. (2000). Transcription of the FMR1 Gene in Individuals with Fragile X Syndrome. Am. J. Med. Genet..

[16] Lévesque S., Dombrowski C., Morel M.-L., Rehel R., Côté J.-S., Bussières J., Morgan K., Rousseau F. (2009). Screening and Instability of FMR1 Alleles in a Prospective Sample of 24,449 Mother–Newborn Pairs from the General Population. Clin. Genet..

[17] Nolin S.L., Glicksman A., Ersalesi N., Dobkin C., Brown W.T., Cao R., Blatt E., Sah S., Latham G.J., Hadd A.G. (2015). Fragile X Full Mutation Expansions Are Inhibited by One or More AGG Interruptions in Premutation Carriers. Genet. Med..

[18] Tassone F., Hagerman R.J., Taylor A.K., Gane L.W., Godfrey T.E., Hagerman P.J. (2000). Elevated Levels of FMR1 MRNA in Carrier Males: A New Mechanism of Involvement in the Fragile-X Syndrome. Am. J. Hum. Genet..

[19] Kenneson A. (2001). Reduced FMRP and Increased FMR1 Transcription Is Proportionally Associated with CGG Repeat Number in Intermediate-Length and Premutation Carriers. Hum. Mol. Genet..

[20] Primerano B., Tassone F., Hagerman R.J., Hagerman P., Amaldi F., Bagni C. (2002). Reduced FMR1 MRNA Translation Efficiency in Fragile X Patients with Premutations. RNA.

[21] Dodds E.D., Tassone F., Hagerman P.J., Lebrilla C.B. (2009). A Polymerase Chain Reaction, Nuclease Digestion, and Mass Spectrometry Based Assay for the Trinucleotide Repeat Status of the Fragile X Mental Retardation 1 Gene. Anal. Chem..

[22] Yuhas J., Walichiewicz P., Pan R., Zhang W., Casillas E.M., Hagerman R.J., Tassone F. (2009). High-Risk Fragile X Screening in Guatemala: Use of a New Blood Spot Polymerase Chain Reaction Technique. Genet. Test. Mol. Biomark..

[23] Tassone F., Iong K.P., Tong T.-H., Lo J., Gane L.W., Berry-Kravis E., Nguyen D., Mu L.Y., Laffin J., Bailey D.B. (2012). FMR1 CGG Allele Size and Prevalence Ascertained through Newborn Screening in the United States. Genome Med..

[24] Zhou Y., Lum J.M., Yeo G.-H., Kiing J., Tay S.K., Chong S.S. (2006). Simplified Molecular Diagnosis of Fragile X Syndrome by Fluorescent Methylation-Specific PCR and GeneScan Analysis. Clin. Chem..

[25] Gatta V., Gennaro E., Franchi S., Cecconi M., Antonucci I., Tommasi M., Palka G., Coviello D., Stuppia L., Grasso M. (2013). MS-MLPA Analysis for FMR1 Gene: Evaluation in a Routine Diagnostic Setting. BMC Med. Genet..

[26] Inaba Y., Schwartz C.E., Bui Q.M., Li X., Skinner C., Field M., Wotton T., Hagerman R.J., Francis D., Amor D.J. (2014). Early Detection of Fragile X Syndrome: Applications of a Novel Approach for Improved Quantitative Methylation Analysis in Venous Blood and Newborn Blood Spots. Clin. Chem..

[27] Kaufmann W.E., Kidd S.A., Andrews H.F., Budimirovic D.B., Esler A., Haas-Givler B., Stackhouse T., Riley C., Peacock G., Sherman S.L. (2017). Autism Spectrum Disorder in Fragile X Syndrome: Cooccurring Conditions and Current Treatment. Pediatrics.

[28] LaFauci G., Adayev T., Kascsak R., Kascsak R., Nolin S., Mehta P., Brown W.T., Dobkin C. (2013). Fragile X Screening by Quantification of FMRP in Dried Blood Spots by a Luminex Immunoassay. J. Mol. Diagn. JMD.

[29] Adayev T., LaFauci G., Dobkin C., Caggana M., Wiley V., Field M., Wotton T., Kascsak R., Nolin S.L., Glicksman A. (2014). Fragile X Protein in Newborn Dried Blood Spots. BMC Med. Genet..

[30] LaFauci G., Adayev T., Kascsak R., Brown W. (2016). Detection and Quantification of the Fragile X Mental Retardation Protein 1 (FMRP). Genes.

[31] Budimirovic D.B., Schlageter A., Filipovic-Sadic S., Protic D.D., Bram E., Mahone E.M., Nicholson K., Culp K., Javanmardi K., Kemppainen J. (2020). A Genotype-Phenotype Study of High-Resolution FMR1 Nucleic Acid and Protein Analyses in Fragile X Patients with Neurobehavioral Assessments. Brain Sci..

[32] Chen L., Yun S.-W., Seto J., Liu W., Toth M. (2003). The Fragile x Mental Retardation Protein Binds and Regulates a Novel Class of MRNAs Containing u Rich Target Sequences. Neuroscience.

[33] Spinner D.S., Kascsak R.B., LaFauci G., Meeker H.C., Ye X., Flory M.J., Kim J.I., Schuller-Levis G.B., Levis W.R., Wisniewski T. (2007). CpG Oligodeoxynucleotide-Enhanced Humoral Immune Response and Production of Antibodies to Prion Protein PrPSc in Mice Immunized with 139A Scrapie-Associated Fibrils. J. Leukoc. Biol..

[34] Kascsak R.J., Fersko R., Pulgiano D., Rubenstein R., Carp R.I. (1997). Immunodiagnosis of Prion Disease. Immunol. Invest..

[35] Chen-Hwang M.-C., Chen H.-R., Elzinga M., Hwang Y.-W. (2002). Dynamin Is a Minibrain Kinase/Dual Specificity Yak1-Related Kinase 1A Substrate. J. Biol. Chem..

[36] Slootstra J.W., Puijk W.C., Ligtvoet G.J., Langeveld J.P.M., Meloen R.H. (1996). Structural Aspects of Antibody-Antigen Interaction Revealed through Small Random Peptide Libraries. Mol. Divers..

[37] Timmerman P., Puijk W.C., Meloen R.H. (2007). Functional Reconstruction and Synthetic Mimicry of a Conformational Epitope Using CLIPS^TM^ Technology. J. Mol. Recognit..

[38] Gholizadeh S., Halder S.K., Hampson D.R. (2015). Expression of Fragile X Mental Retardation Protein in Neurons and Glia of the Developing and Adult Mouse Brain. Brain Res..

[39] Arsenault J., Gholizadeh S., Niibori Y., Pacey L.K., Halder S.K., Koxhioni E., Konno A., Hirai H., Hampson D.R. (2016). FMRP Expression Levels in Mouse Central Nervous System Neurons Determine Behavioral Phenotype. Hum. Gene Ther..

[40] Petkov P.M., Ding Y., Cassell M.A., Zhang W., Wagner G., Sargent E.E., Asquith S., Crew V., Johnson K.A., Robinson P. (2004). An Efficient SNP System for Mouse Genome Scanning and Elucidating Strain Relationships. Genome Res..

[41] Shimada A. (1999). Age-Dependent Cerebral Atrophy and Cognitive Dysfunction in SAMP10 Mice. Neurobiol. Aging.

[42] Yan Q.J., Rammal M., Tranfaglia M., Bauchwitz R.P. (2005). Suppression of Two Major Fragile X Syndrome Mouse Model Phenotypes by the MGluR5 Antagonist MPEP. Neuropharmacology.

[43] Musumeci S.A., Bosco P., Calabrese G., Bakker C., De Sarro G.B., Elia M., Ferri R., Oostra B.A. (2000). Audiogenic Seizures Susceptibility in Transgenic Mice with Fragile X Syndrome. Epilepsia.

[44] Musumeci S.A., Calabrese G., Bonaccorso C.M., D’Antoni S., Brouwer J.R., Bakker C.E., Elia M., Ferri R., Nelson D.L., Oostra B.A. (2007). Audiogenic Seizure Susceptibility Is Reduced in Fragile X Knockout Mice after Introduction of FMR1 Transgenes. Exp. Neurol..

[45] Bonaccorso C.M., Spatuzza M., Di Marco B., Gloria A., Barrancotto G., Cupo A., Musumeci S.A., D’Antoni S., Bardoni B., Catania M.V. (2015). Fragile X Mental Retardation Protein (FMRP) Interacting Proteins Exhibit Different Expression Patterns during Development. Int. J. Dev. Neurosci. Off. J. Int. Soc. Dev. Neurosci..

[46] White J.R., Gong H., Colaizy T., Moreland J.G., Flaherty H., McElroy S.J. (2016). Evaluation of Hematologic Composition in Newborn C57/BL6 Mice up to Day 35. Vet. Clin. Pathol. Am. Soc. Vet. Clin. Pathol..

[47] Dos Santos Pessini P.G., de Souza P.R.K., dos Santos Chagas C., Sampaio E.G., Neves D.S., Petri G., Fonseca F.L.A., da Silva E.B. (2020). Hematological Reference Values and Animal Welfare Parameters of BALB/C-FMABC (Mus Musculus) Inoculated with Ehrlich Tumor Kept in the Vivarium at ABC Medical School. Anim. Models Exp. Med..

[48] Silva-Santana G., Bax J.C., Fernandes D.C.S., Bacellar D.T.L., Hooper C., Dias A.A.S.O., Silva C.B., de Souza A.M., Ramos S., Santos R.A. (2020). Clinical Hematological and Biochemical Parameters in Swiss, BALB/c, C57BL/6 and B6D2F1 Mus Musculus. Anim. Models Exp. Med..

