# Supplementary material for: Development of a Quantitative FMRP Assay for Mouse Tissue Applications
Source: Genes (Basel). 2021 Sep 26;12(10):1516. doi: 10.3390/genes12101516 (PMC8535242; doi:10.3390/genes12101516)
Supplement: Supplementary file 1 [file genes-12-01516-s001.zip › Supplementary Materials_ Report S1_Pepscan.pdf]

Supplementary Materials

Report S1:
Summary of the precision epitope mapping data for anti-mouse FMRP and anti-human FMRP antibody clones.

Linear peptide analysis was performed by the Pepscan Presto BV using 15-mer peptides from amino acids 50-500 of human FMRP. It was used to determine the exact linear epitopes for the total of 16 FMRP specific mAbs. The pepscans and epitopes for the 14 antibodies are shown below: nine derived from immunization with mouse FMRP and five derived from immunization with human FMRP. This is in addition to the two clones previously mapped (mAb 6B8 epitope aa345-350, $_{345}$**VGPNAP**$_{350}$, and 5C2 aa347-353).

A. ***The major region of response for the mouse FMRP immunogen***

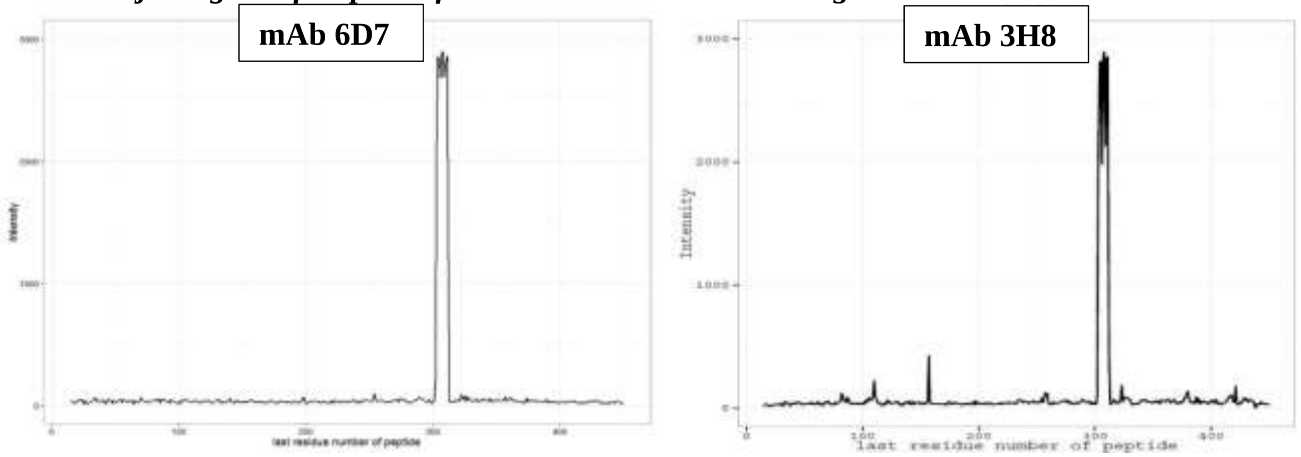

The largest set of mAbs from the fusion using mouse FMRP revealed an epitope $_{347}$**PNAPEEK**$_{353}$ reactive to human and mouse FMRP. This includes clones 1E3, 1B12, 5C2, 3D2, 6C2, 6D7 and 6E4 that shared the same epitope (graph of mAb 6D7 is shown as an example of this entire group). One additional clone 3H8 from the mouse immunogen revealed a very similar epitope site, **aa 346-355**, a single amino acid change at the N terminal site. This reinforces the observation that this was the major region of response for the mouse FMRP immunogen and further emphasizes the difference in response between the human and mouse immunogens.

B. ***The major region of response for the human FMRP immunogen***

The results using the human FMRP immunogen was quite different. The Pepscan for clones 2E5 and 2B10 are shown below. The epitope is $_{418}$**DYHLNYLKEV**$_{427}$. This epitope is very similar to another clone derived from the human FMRP fusion, clones 2D10, epitope $_{417}$**LDYHLNKLKE**$_{426}$. While, this site is clearly a dominant region for the human FMRP, it was not recognized in the mouse immunized with mouse FMRP.

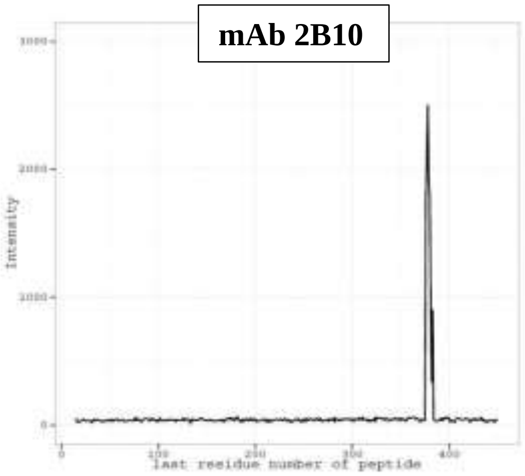

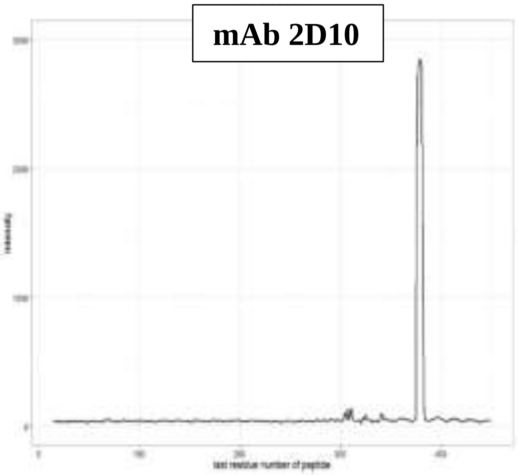

C. ***The clones with N terminal epitopes.***

The epitopes for the human FMRP derived clone 2G5 and mouse FMRP derived clone 10H12 are very similar (see below). Another clone with a similar epitope is the human FMRP derived clone 3E7a the epitope for this clone is $_{120}$**PATKDTFH**$_{127}$

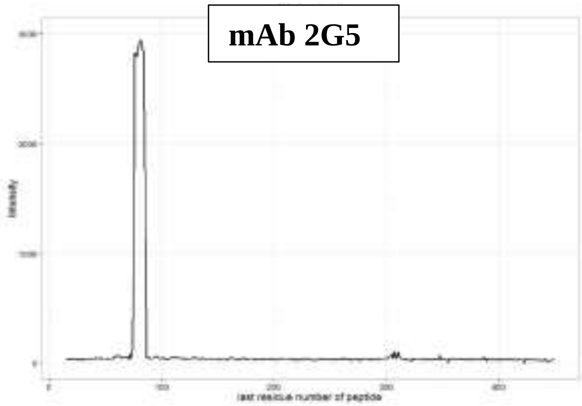

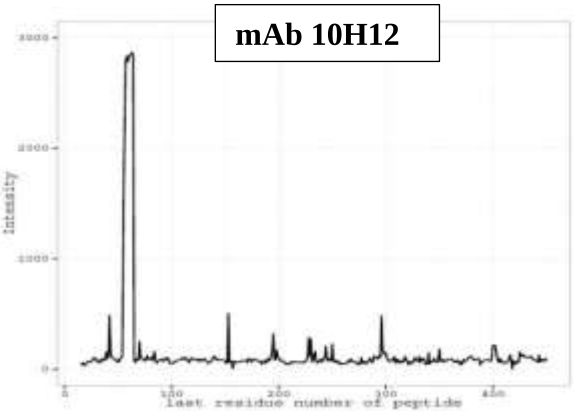

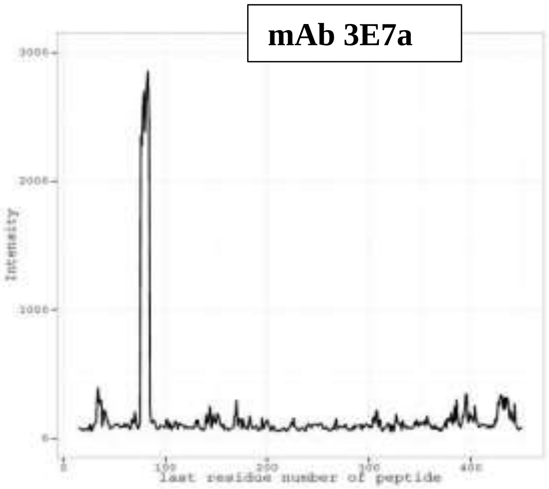

3E7a (human) $_{120}$**PATKDTFH**$_{127}$
2G5 (human) $_{120}$**PATKDTFH**$_{127}$
10H12 (mouse) $_{99}$**CDATYNEI**$_{107}$

D. ***The clones with C terminal epitopes.***

The antibody mAb 7D9 maps to a C terminal region $_{470}$**GRGSRPYR**$_{477}$ derived from the mouse FMRP immunogen. This clone is the most C terminal of all the FMRP antibodies mapped by Pepscan.

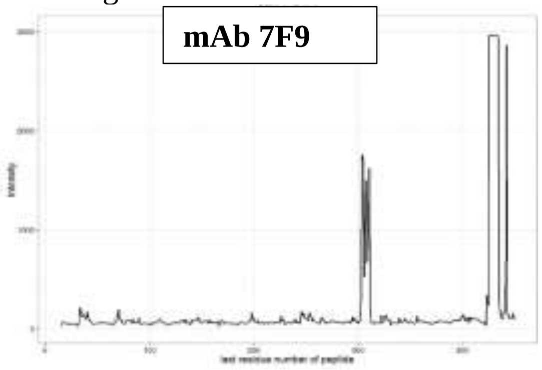

The 3D structural analysis of select FMRP antibody clones

The Pepscan provided a 3D structural analysis of the sites recognized by several of the FMRP antibody clones. The human clones 2G5 and 2D10 are shown below. Epitopes identified in Pepscan were visualized. They occupy solvent exposed structural elements of the target structure. This may indicate these regions as hydrophilic and amenable to immune response.

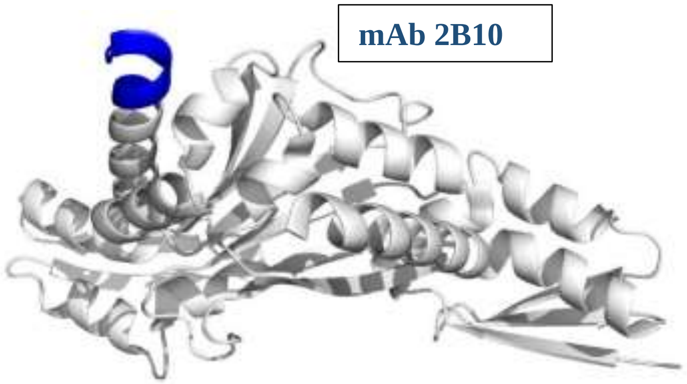

Top panel:
The 3D ribbon structure of FMRP aa 266-475 is rendered in grey cartoon with peptide stretch $_{418}$***DYHLNYLKEV***$_{427}$ highlighted in blue.

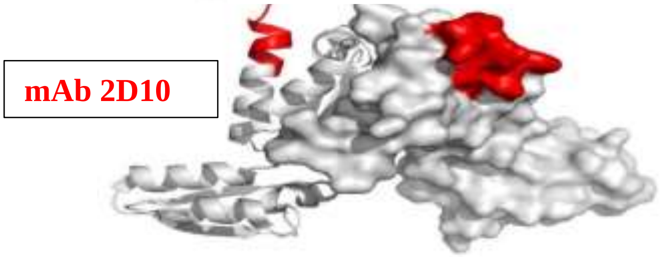

Middle panel:
The 3D site for 2D10 is shown in white cartoon with the peptide stretch $_{417}$***LDYHLNYLKE***$_{426}$ highlighted in red.

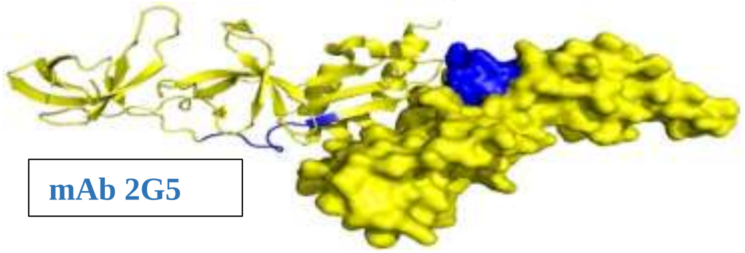

Bottom panel:
The 3D structure of FMRP fragment aa 50 - 263 is rendered in yellow cartoon with the peptide stretch $_{120}$***PATKDTFH***$_{127}$ and the site for 2G5 is shown in blue.
