# Supplementary material for: Development of a Quantitative FMRP Assay for Mouse Tissue Applications
Source: Genes (Basel). 2021 Sep 26;12(10):1516. doi: 10.3390/genes12101516 (PMC8535242; doi:10.3390/genes12101516)
Supplement: Supplementary file 1 [file genes-12-01516-s001.zip › Supplementary Materials_Figure S1.pdf]

genes

Supplementary Materials:

Figure S1

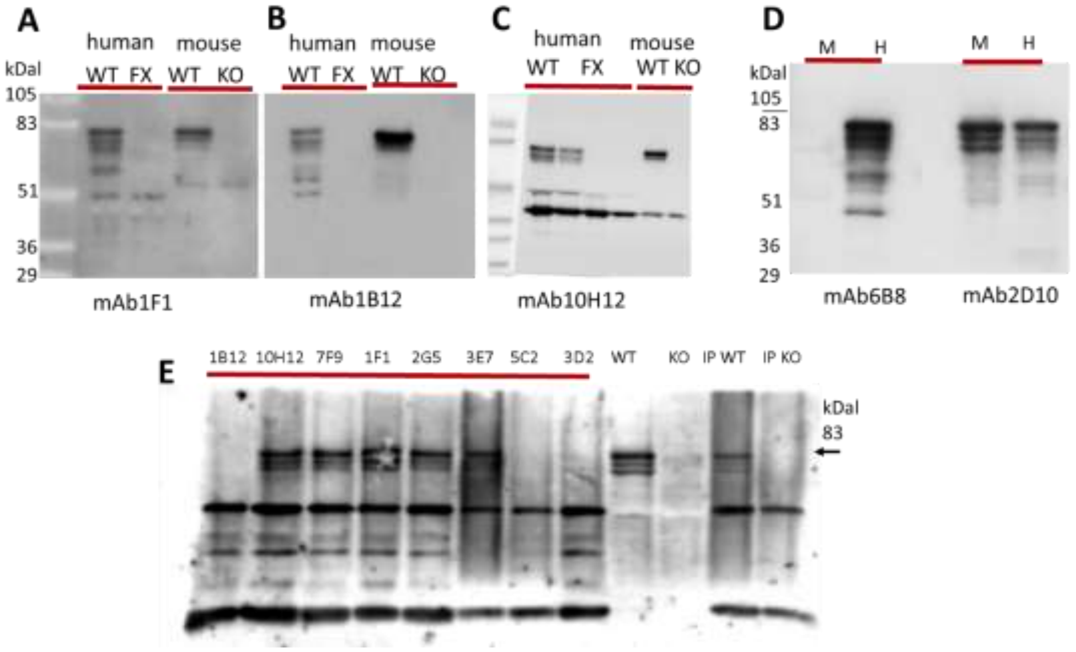

Figure S1. Western blot analysis and immunoprecipitation studies for select mAb clones.

A, B, C: Western Blot with mAb1F1 (A) and mAb1B12 (B) and mAb10H12 (C) of human lymphoblastoid cell lines (LTL) from a normal individual (WT), a male FM (FX), and 2) WT and KO mouse brains (WT). Each well was loaded with 15 mg of sample (except for (C) where 30 and 15 mg of LTL lysates were loaded respectively)

D: Western Blot analysis with mAb6B8 and mAb2D10 of normal human lymphoblastoid cells and of a WT mouse brain homogenate. Each gel lane was loaded with 15 ug of sample.

E: Competitive immunoprecipitation. Mouse WT brain samples were immunoprecipitated with mAb1B12 in presence of an excess of an unbound antibody (indicated on top of each lane). Lanes labelled WT, and KO were loaded with WT and KO brain homogenates, respectively. Immunoprecipitation of homogenates from WT and KO mouse with mAb1B12 in absence of a competitor antibody are indicated IPWT and IPKO.
